# Unusual Presentation of Odontogenic Cellulitis Mimicking Drug-Induced Angioedema: A Case Report

**DOI:** 10.7759/cureus.95162

**Published:** 2025-10-22

**Authors:** P. Bal Reddy, Syed Humayun, D. Naga Sujata, K. Anil Kumar, J. Sanjeev Naik

**Affiliations:** 1 Oral and Maxillofacial Surgery, Government Dental College & Hospital, Hyderabad, IND; 2 Oral and Maxillofacial Surgery, Prince Saud Bin Jalawy (PSBJ) Hospital, Al Ahsa, SAU; 3 Oral and Maxillofacial Surgery, Government Dental College & Hospital, Vijayawada, IND; 4 Oral and Maxillofacial Surgery, Government Medical College, Nandyal, IND; 5 Oral and Maxillofacial Surgery, Gandhi Hospital, Secunderabad, IND

**Keywords:** angioedema, facial swelling, ibuprofen, non-vital tooth, odontogenic cellulitis, periapical cyst

## Abstract

Facial cellulitis secondary to odontogenic infections can present with clinical features that closely mimic drug-induced angioedema, leading to diagnostic confusion and delayed appropriate treatment. We report a 23-year-old female who presented with rapid-onset bilateral midfacial swelling following her first exposure to ibuprofen. Initial clinical assessment by a general practitioner suggested drug-induced angioedema, and the patient was treated with antihistamines and corticosteroids. However, a detailed dental examination revealed a non-vital maxillary lateral incisor with associated periapical pathology. Clinical features, including erythema, warmth, and tenderness, along with laboratory findings demonstrating neutrophilia without eosinophilia and radiographic evidence of periapical infection, ultimately confirmed the diagnosis of odontogenic cellulitis rather than allergic angioedema. The patient was admitted and treated with empirical broad-spectrum intravenous antibiotics, followed by surgical drainage and extraction of the offending tooth, resulting in complete resolution within seven days. Definitive treatment with cyst enucleation was performed one week post-discharge with satisfactory outcomes. This case emphasizes the critical importance of comprehensive dental evaluation in patients presenting with bilateral facial swelling, even when drug exposure temporally correlates with symptom onset, as proper differential diagnosis prevents inappropriate treatment and ensures optimal patient outcomes.

## Introduction

Facial cellulitis represents a serious soft tissue infection characterized by acute, painful, spreading inflammation of the subcutaneous and deeper fascial spaces [1]. Odontogenic infections account for approximately 10-12% of all facial cellulitis cases, with the midface region being particularly vulnerable due to the proximity of dental apices to fascial planes [2]. These infections commonly create diffuse erythematous swelling that may extend to periorbital areas, and when bilateral involvement occurs, the clinical presentation can closely mimic non-infectious conditions such as angioedema [3].

Angioedema, conversely, is a non-inflammatory condition characterized by rapid-onset, asymmetric swelling of the deeper dermis and subcutaneous tissues, most commonly affecting the face, lips, and eyelids [4]. Drug-induced angioedema, particularly from nonsteroidal anti-inflammatory drugs (NSAIDs), represents a well-recognized clinical entity that can occur within hours of medication exposure [5]. The incidence of NSAID-induced angioedema ranges from 0.1% to 0.3% of exposed individuals, with higher rates in patients with underlying allergic conditions [6].

The diagnostic challenge arises when odontogenic cellulitis and drug-induced angioedema present with overlapping clinical features, particularly bilateral facial swelling with temporal association to medication use. Misdiagnosis can lead to inappropriate treatment, delayed resolution, and potential life-threatening complications, including airway compromise or sepsis [7]. This diagnostic confusion is particularly relevant given that patients with dental pain often self-medicate with NSAIDs prior to seeking care, creating an apparent causal relationship between drug exposure and facial swelling.

We present a case of odontogenic cellulitis secondary to an infected periapical cyst that was initially misdiagnosed as ibuprofen-induced angioedema, highlighting the critical importance of comprehensive clinical evaluation, systematic differential diagnosis, and the integration of laboratory and radiographic findings in clinical decision-making.

## Case presentation

Patient information and presenting complaint

A 23-year-old female presented to the Department of Oral and Maxillofacial Surgery with a chief complaint of rapidly progressive bilateral midfacial swelling of one day's duration. The patient reported taking a single 400 mg ibuprofen tablet for a mild headache approximately 24 hours prior to presentation, representing her first documented exposure to this medication.

Clinical history

The patient noted that facial swelling began approximately two hours after ibuprofen ingestion, initially localized to the upper lip before progressing to involve bilateral labial, buccal, and periorbital regions within 12 hours. She had initially consulted a general medical practitioner who diagnosed drug-induced angioedema based on the temporal relationship to medication exposure and prescribed oral antihistamines (cetirizine 10 mg) and corticosteroids (prednisolone 20 mg). When symptoms failed to improve after 12 hours of treatment, a dental consultation was sought.

The patient's medical history was unremarkable, with no known drug allergies, previous episodes of angioedema, hereditary angioedema, or systemic diseases. She denied fever, difficulty breathing, dysphagia, or urticaria. There was no history of recent dental procedures or trauma.

Physical examination

**Figure 1:**
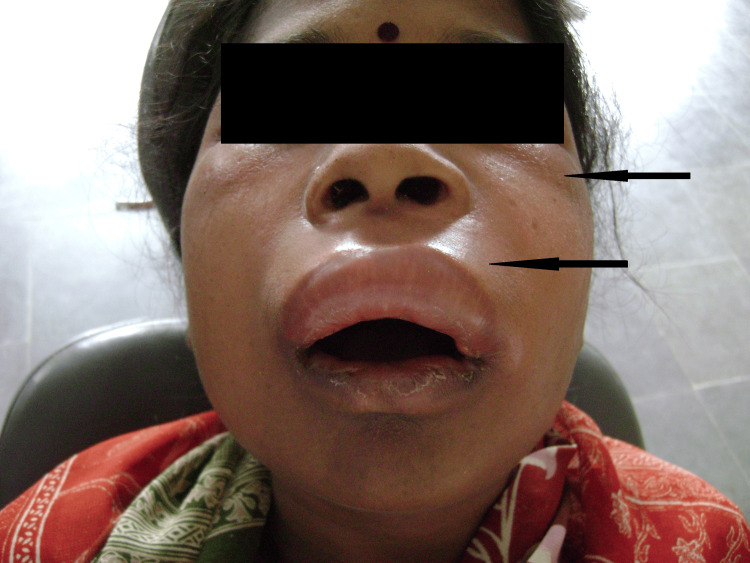
Initial clinical presentation on admission showing bilateral midfacial and periorbital swelling with complete bilateral eyelid closure. The erythematous and warm overlying skin (indicated by arrows) distinguishes this acute infectious process from typical angioedema, which characteristically presents with non-erythematous, non-tender swelling.

Physical examination revealed a moderately distressed patient with significant bilateral midfacial swelling. The swelling measured approximately 8×10 cm (measured horizontally over the upper lip from side to side and diagonally from lateral canthus to upper lip vermillion), involving the labial, buccal, canine, and periorbital spaces bilaterally, resulting in complete eyelid closure (Figure 1). The overlying skin was erythematous, warm to the touch, and mildly tender on palpation-features consistent with inflammation. The swelling demonstrated firm consistency without fluctuation or crepitus.

Vital signs were within normal limits: blood pressure 118/74 mmHg, pulse 84 beats per minute, respiratory rate 18 breaths per minute, and temperature 98.6°F (37°C). The patient maintained normal respiratory function with clear breath sounds and preserved airway patency. Swallowing ability was intact. No cervical lymphadenopathy was detected on palpation. Visual acuity was preserved bilaterally, though extraocular movements were restricted due to periorbital edema.

Intraoral examination

**Figure 2:**
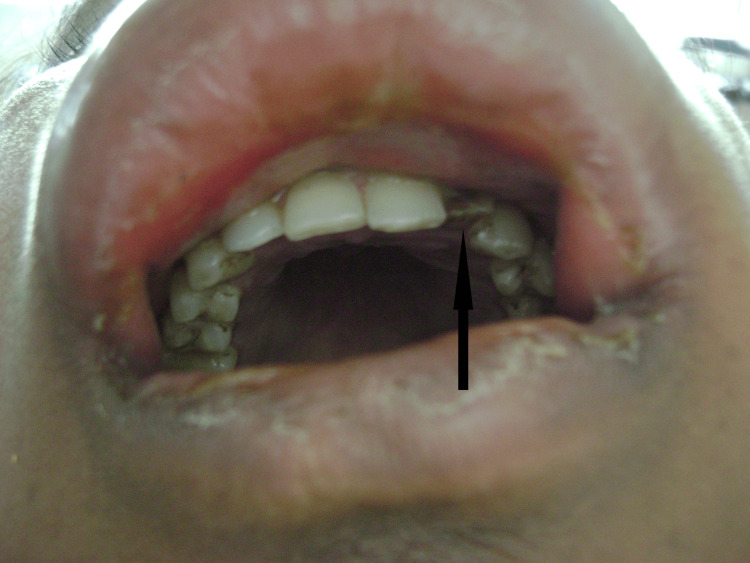
Intraoral photograph demonstrating the discolored, non-vital maxillary left lateral incisor with fractured crown (black arrow) identified as the primary source of odontogenic infection. Note the visible discoloration of the affected tooth.

Intraoral examination revealed a non-vital maxillary left lateral incisor with gray discoloration and a fractured crown involving approximately 40% of the clinical crown, non-responsive to thermal and electric pulp testing (Figure 2). The tooth exhibited grade I mobility. The labial vestibule adjacent to tooth #22 appeared obliterated by swelling and was unexaminable. Mild gingival inflammation was noted around the affected tooth. No obvious dental caries or periodontal pathology was observed in adjacent teeth. Oral hygiene was fair.

Differential diagnosis and clinical reasoning

Based on clinical presentation and history, the differential diagnosis included drug-induced angioedema supported by temporal relationship to ibuprofen exposure, bilateral distribution, and first exposure to medication; however, argued against by presence of erythema, warmth, tenderness, and absence of urticaria or systemic allergic symptoms. Odontogenic cellulitis is suggested by the presence of a non-vital tooth with periapical pathology, inflammatory signs (erythema, warmth, tenderness), firm swelling, and failure to respond to antihistamines. Hereditary angioedema is less likely given no family history and the presence of inflammatory signs. Acute allergic reaction unlikely due to absence of urticaria, pruritus, or systemic symptoms.

Diagnostic investigations

Laboratory Studies

A complete blood count revealed leukocytosis (white blood cell count: 12,400/μL, normal range: 4,000-11,000/μL) with neutrophilia (neutrophil count: 8,200/μL, 66%, normal range: 1,800-7,000/μL) without eosinophilia (eosinophil count: 180/μL, 1.5%, normal range: 15-500/μL). C-reactive protein was elevated at 45 mg/L (normal <10 mg/L). The absence of eosinophilia argued strongly against allergic etiology, while neutrophilia and elevated inflammatory markers supported an acute infectious process [8].

Radiographic Assessment

**Figure 3:**
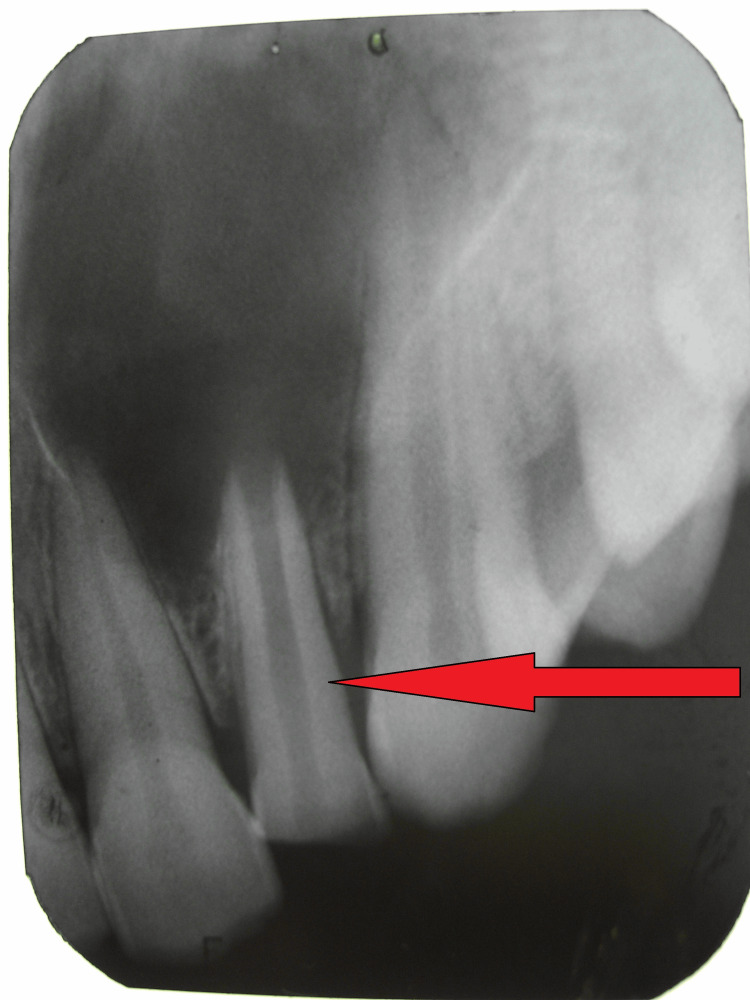
Periapical radiograph revealing ill-defined radiolucency associated with the fractured maxillary left lateral incisor (red arrow), consistent with infected periapical cyst formation.

Intraoral periapical radiography demonstrated an open apex with incomplete root development and a well-defined periapical radiolucency measuring approximately 2×3 cm associated with the maxillary left lateral incisor, with extension toward the adjacent central incisor and canine (Figure 3). Loss of lamina dura and cortical bone erosion were evident. The radiographic appearance was consistent with an infected periapical cyst of dental origin.

Integration of Findings

The combination of inflammatory clinical signs, neutrophilia without eosinophilia, radiographic evidence of periapical pathology, and lack of response to antihistamines established the diagnosis of odontogenic cellulitis rather than drug-induced angioedema. The temporal relationship to ibuprofen use was deemed coincidental, likely representing the patient's attempt to self-medicate preexisting dental pain.

Treatment and management

Initial Management: Day One

The patient was admitted for close monitoring and empirical broad-spectrum antibiotic therapy. Initial treatment included intravenous cefotaxime 1 g twice daily (third-generation cephalosporin providing coverage against *streptococci* and oral anaerobes), gentamicin 80 mg three times daily (aminoglycoside for additional gram-negative coverage), and metronidazole 500 mg three times daily (specific anaerobic coverage) [9]. This triple-antibiotic regimen was selected to provide comprehensive coverage of mixed oral flora commonly implicated in odontogenic infections, including *Streptococcus viridans*, *Peptostreptococcus*, *Prevotella*, and *Fusobacterium* species.

Given initial diagnostic uncertainty, hydrocortisone 50 mg four times daily and pheniramine maleate 10 mg twice daily were continued for the first 24 hours to cover the possibility of angioedema, with plans to discontinue if clinical improvement occurred with antibiotics.

Clinical course and definitive treatment

Day One and Two

**Figure 4:**
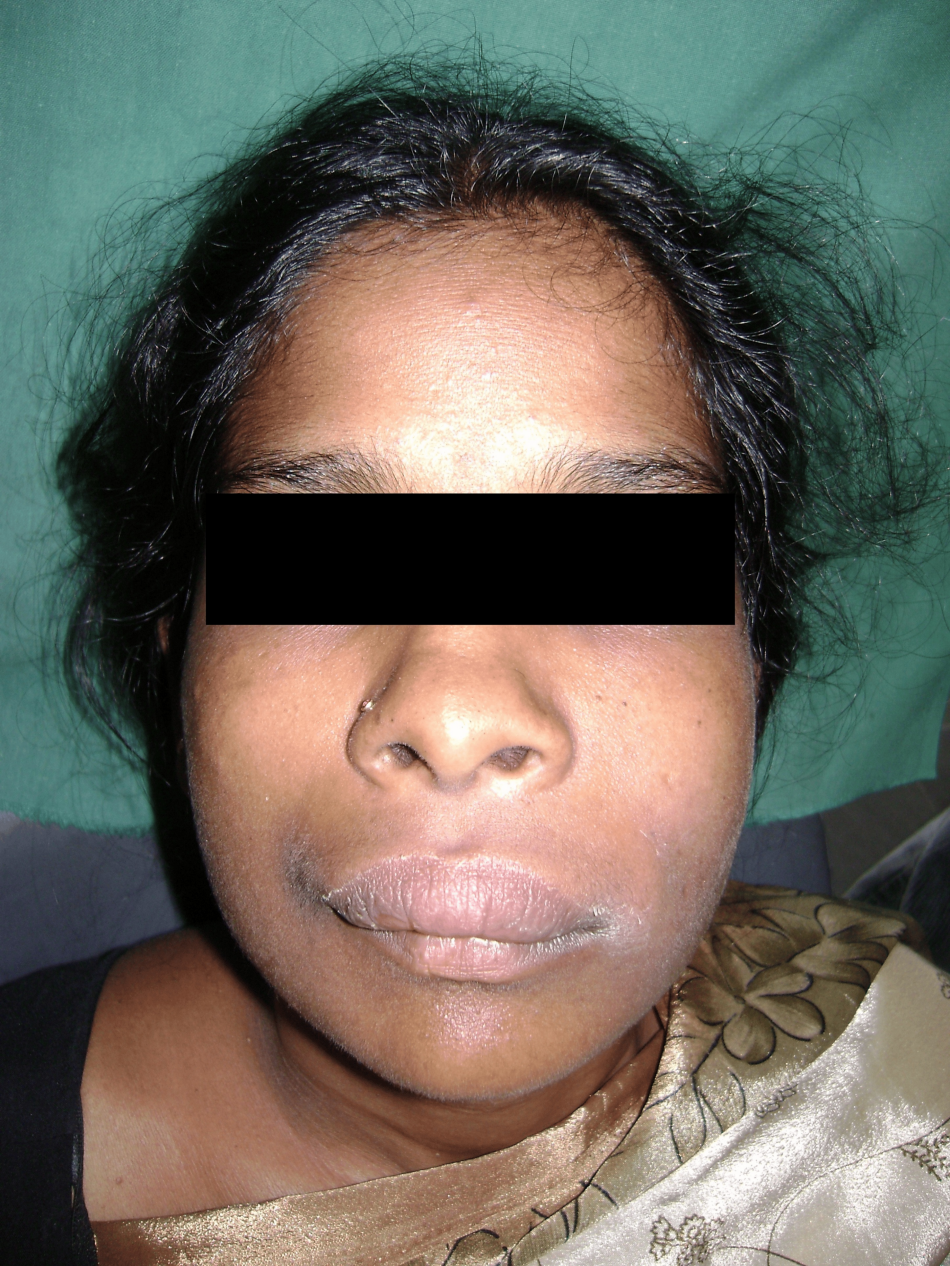
Clinical photograph on hospital day two, demonstrating marked reduction in bilateral facial swelling and decreased erythematous changes following initiation of intravenous antibiotic therapy.

Significant clinical improvement was observed within 24 hours, with a marked reduction in facial swelling (approximately 40% decrease) and decreased erythema (Figure 4). Antihistamines and corticosteroids were discontinued as allergic etiology was definitively ruled out based on treatment response.

Day Three

**Figure 5:**
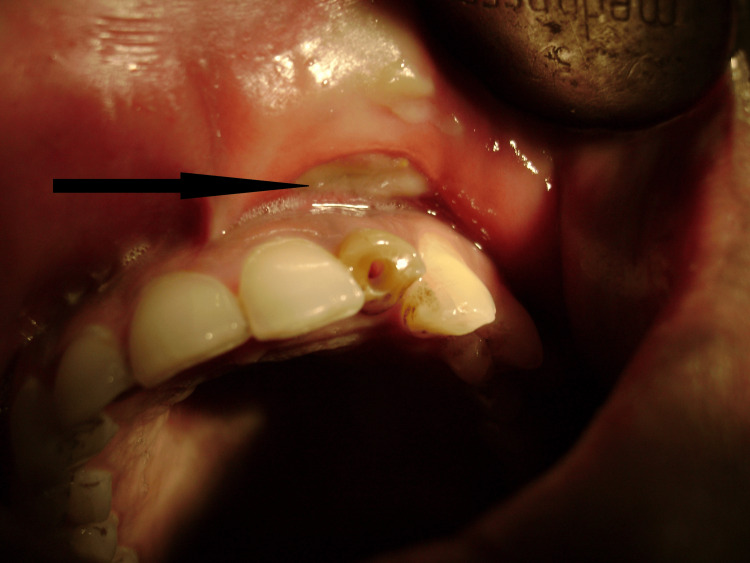
Intraoral view following incision and drainage procedure performed on hospital day three. The drainage site (black arrow) is visible in the upper labial vestibule adjacent to the affected tooth, with active purulent drainage noted at the time of the procedure.

Intraoral swelling developed with pointing in the upper labial vestibule adjacent to tooth #22. Incision and drainage were performed under local anesthesia (2% lidocaine with 1:100,000 epinephrine), yielding approximately 5 ml of purulent material that was submitted for bacterial culture and antibiotic sensitivity testing (Figure 5).

Day Four

An access cavity was prepared in the maxillary left lateral incisor under rubber dam isolation to establish endodontic drainage and decompress the periapical infection. Copious irrigation with normal saline was performed.

Day Five

Extraction of the non-vital maxillary left lateral incisor was performed under local anesthesia due to poor prognosis (fractured crown, open apex, large periapical lesion). The socket was irrigated with normal saline. Culture results confirmed mixed gram-positive cocci (*Streptococcus viridans* group) and anaerobes (*Peptostreptococcus* species) sensitive to the prescribed antibiotic regimen.

Day Seven

**Figure 6:**
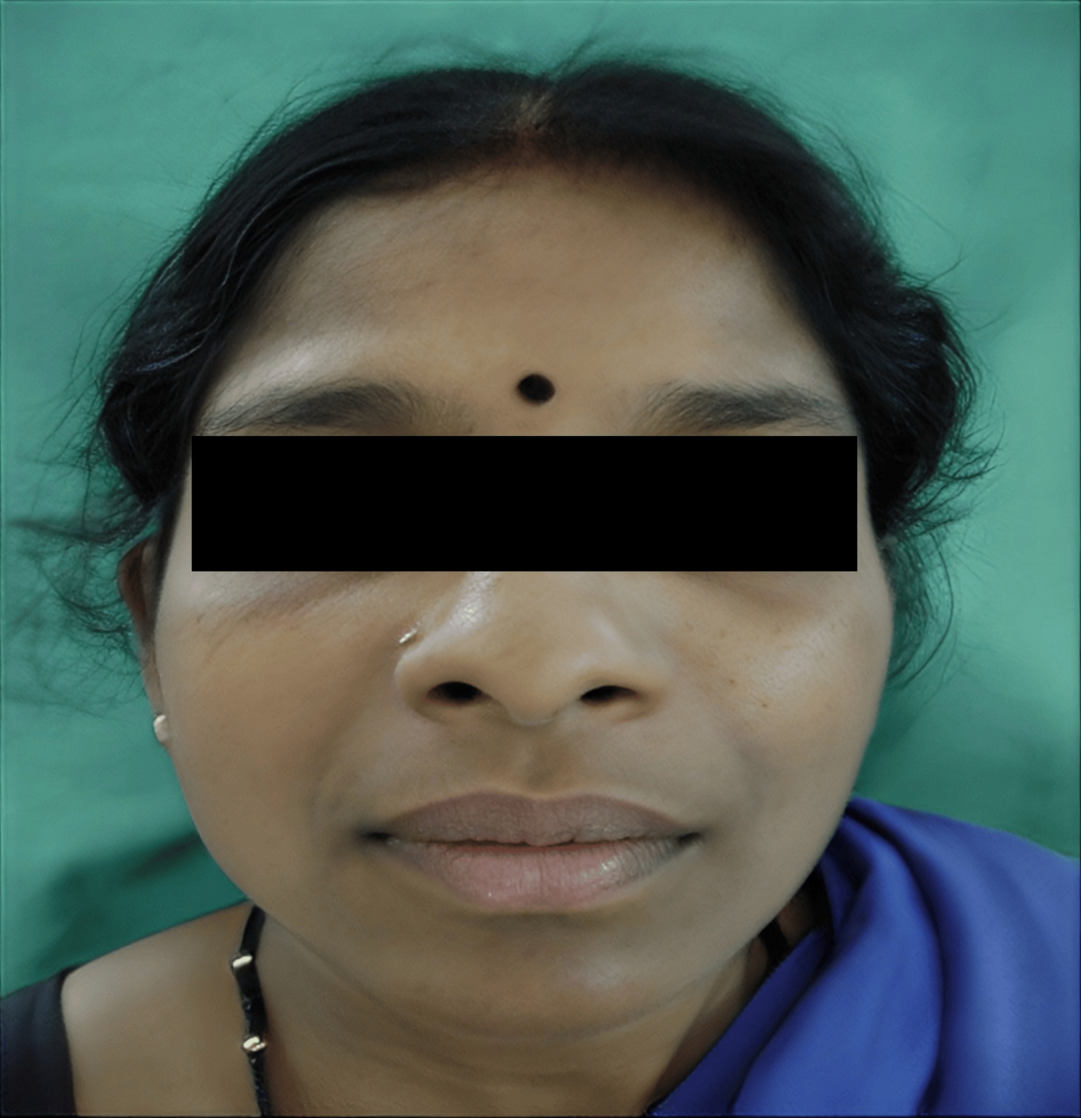
Clinical appearance at hospital discharge on day seven, showing complete resolution of facial swelling and erythema.

Complete resolution of facial swelling was achieved with restoration of normal facial contours and resolution of erythema. The patient was discharged with oral antibiotics (amoxicillin-clavulanate 625 mg three times daily for five days) and analgesics (Figure 6). 

Definitive management of residual pathology

**Figure 7:**
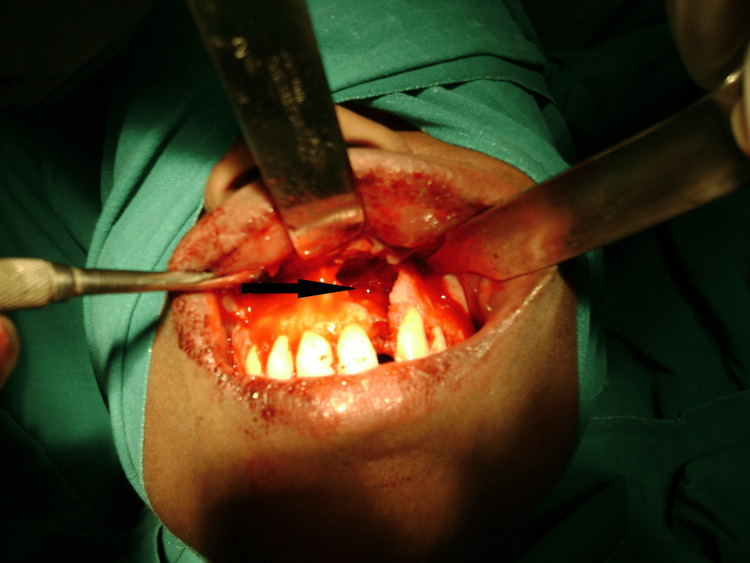
Intraoperative photograph obtained during surgical enucleation showing the cystic cavity (black arrow) following complete removal of the periapical lesion.

**Figure 8:**
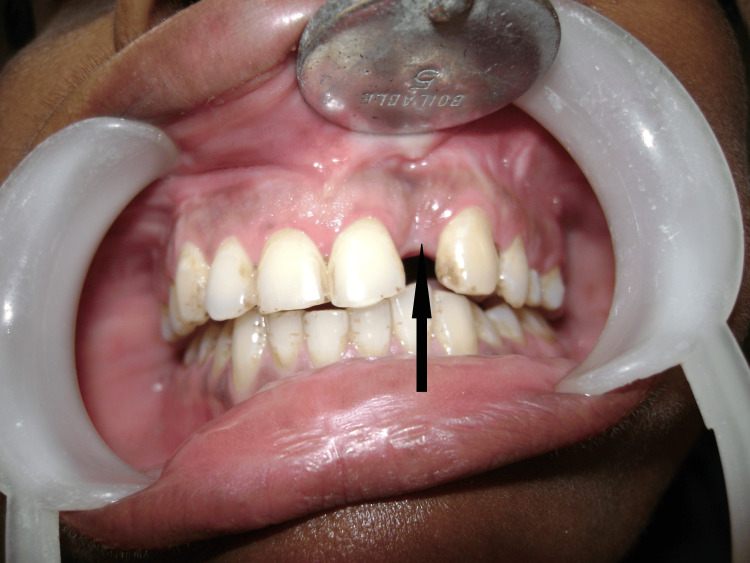
Intraoral photograph at two-week postoperative follow-up visits demonstrating satisfactory healing of both the extraction socket and surgical site (black arrow) with no signs of infection or delayed healing.

One week following discharge, the patient returned for definitive management of the residual periapical cyst. Under local anesthesia, complete enucleation of the cystic lesion measuring 2.5×2 cm was performed via a labial approach with primary closure using 3-0 Vicryl sutures (Figures 7, 8). The specimen was submitted for histopathological examination, which confirmed the diagnosis of an infected periapical (radicular) cyst with chronic inflammatory cell infiltrate.

Follow-up and outcome

A two-week follow-up revealed satisfactory healing with complete mucosal re-epithelialization and no complications. Sutures had dissolved appropriately. The patient remained asymptomatic at three-month follow-up with no recurrence of swelling, infection, or functional impairment.

## Discussion

This case illustrates the diagnostic challenge posed when odontogenic cellulitis presents with clinical features suggestive of drug-induced angioedema. Several factors contributed to the initial misdiagnosis, including the temporal relationship between ibuprofen exposure and symptom onset, bilateral distribution of swelling, and the patient's first documented exposure to the medication. However, systematic clinical evaluation with integration of laboratory and radiographic findings successfully identified the correct diagnosis (Table 1).

**Table 1:** Differential features of cellulitis vs. angioedema

Feature	Odontogenic Cellulitis	Drug-Induced Angioedema
Onset	Gradual (hours to days)	Rapid (minutes to hours)
Skin changes	Erythematous, warm	Pale, normal temperature
Tenderness	Present, painful	Minimal or absent
Consistency	Firm, indurated	Soft, pitting
Distribution	Follows fascial spaces	Non-anatomical
Associated findings	Dental pathology, fever	Urticaria, pruritus
Laboratory	Neutrophilia, elevated CRP	Eosinophilia (may be present)
Response	Antibiotics + drainage	Antihistamines, corticosteroids

Key clinical features that ultimately favored cellulitis over angioedema included the presence of inflammatory signs such as erythema, warmth, and tenderness, which strongly suggested an infectious etiology [10]. Laboratory findings revealed neutrophilia with elevated inflammatory markers but without eosinophilia, supporting bacterial infection rather than allergic reaction [8]. Radiographic evidence demonstrated clear periapical pathology, providing definitive evidence of an odontogenic source. Furthermore, the patient's rapid improvement with antibiotics rather than antihistamines confirmed the diagnosis of cellulitis.

The maxillary anterior region lacks significant anatomical barriers to infection spread, allowing periapical infections to disseminate rapidly into multiple fascial spaces, including the canine space, buccal space, and periorbital tissues [11]. The close proximity of the canine fossa to the infraorbital area explains the characteristic pattern of swelling observed in this case. The bilateral presentation likely resulted from midline extension through loose areolar tissue planes.

NSAIDs represent a significant cause of pseudoallergic angioedema, with aspirin being the most frequently implicated agent, followed by ibuprofen, naproxen, and diclofenac. These reactions result from cyclooxygenase inhibition, which redirects arachidonic acid metabolism through the lipoxygenase pathway, leading to increased synthesis of cysteinyl leukotrienes and other vasoactive mediators [12]. NSAID-induced angioedema typically manifests within 30 minutes to 2 hours of drug exposure and presents with non-erythematous, soft swelling that may be associated with urticarial lesions and pruritus [13]. Diagnosis relies primarily on clinical history and exclusion of other causes. The absence of these typical angioedema features in our patient, combined with the presence of inflammatory signs and lack of eosinophilia, effectively ruled out this diagnosis.

The initial empirical approach addressing both differential diagnoses was appropriate, given the diagnostic uncertainty and potential severity of both conditions. Early antibiotic therapy with broad-spectrum coverage targeting oral flora, including anaerobic organisms, represents standard care for odontogenic infections [9]. Surgical intervention, including drainage and source control through tooth extraction, is essential for the resolution of established odontogenic infections with abscess formation [14]. The staged approach used in this case, with initial drainage followed by definitive cyst enucleation after resolution of acute infection, ensured complete treatment while minimizing surgical morbidity during the acute inflammatory phase.

Limitations: This case report represents a single patient experience and is subject to limitations inherent in retrospective case reporting. Formal allergy testing with oral challenge was not performed to definitively exclude ibuprofen sensitivity, though the clinical and laboratory evidence strongly argued against this diagnosis. Additionally, microbiological culture was obtained only after incision and drainage rather than at initial presentation.

## Conclusions

Odontogenic cellulitis can closely mimic drug-induced angioedema, particularly when bilateral facial involvement occurs in temporal association with medication exposure. This case highlights several critical learning points for clinical practice. First, the presence of inflammatory signs, including erythema, warmth, and tenderness, strongly favors cellulitis over angioedema and should prompt investigation for infectious sources. Second, laboratory differentiation using complete blood count with differential and inflammatory markers effectively guides diagnosis, with neutrophilia supporting infection and eosinophilia suggesting allergy. Third, radiographic evaluation is essential to identify underlying dental pathology in patients presenting with facial swelling. Fourth, the temporal relationship between medication use and symptom onset may be coincidental rather than causal, particularly when patients self-medicate for dental pain. Finally, prompt recognition and appropriate multidisciplinary management of odontogenic infections prevent serious complications, including airway compromise, orbital involvement, and descending necrotizing mediastinitis.

Healthcare providers should maintain high clinical suspicion for odontogenic sources of facial swelling, even when alternative diagnoses appear likely based on history alone. Comprehensive clinical evaluation, including dental assessment, systematic integration of laboratory and imaging findings into diagnostic reasoning, and appropriate empirical therapy addressing the most likely diagnoses, ensures optimal patient outcomes. Early recognition, appropriate surgical source control, and targeted antibiotic therapy remain the cornerstones of successful management of odontogenic infections.
